# Agreements and controversies of national guidelines for bronchiolitis: Results from an Italian survey

**DOI:** 10.1002/iid3.451

**Published:** 2021-10-22

**Authors:** Sara Manti, Amelia Licari, Ilaria Brambilla, Carlo Caffarelli, Mauro Calvani, Fabio Cardinale, Giorgio Ciprandi, Claudio Cravidi, Marzia Duse, Alberto Martelli, Domenico Minasi, Michele Miraglia Del Giudice, Giovan B. Pajno, Maria A. Tosca, Elena Chiappini, Eugenio Baraldi, Gianluigi Marseglia

**Affiliations:** ^1^ Dipartimento di Medicina Clinica e Sperimentale Unità di Broncopneumologia Pediatrica, Università di Catania Catania Italy; ^2^ Clinica Pediatrica, Fondazione IRCCS Policlinico “S. Matteo,” Università di Pavia Pavia Italy; ^3^ Clinica Pediatrica, Dipartimento di Medicina e Chirurgia Università di Parma Parma Italy; ^4^ Dipartimento di Pediatria Ospedale S. Camillo‐Forlanini Roma Italy; ^5^ UOC Pediatria, Servizio di Allergologia e Pneumologia Pediatrica, Azienda Ospedaliera‐Universitaria “Consorziale‐Policlinico,” Ospedale Pediatrico Giovanni XXIII Bari Italy; ^6^ Casa di Cura Villa Montallegro Genoa Italy; ^7^ Family Paediatrics, ATS Pavia Pavia Italy; ^8^ Dipartimento di Pediatria, Policlinico Umberto I, Università Sapienza di Roma Roma Italy; ^9^ UOC Pediatria, Azienda Ospedaliera G. Salvini, Ospedali di Garbagnate Milanese e Bollate Milano Italy; ^10^ UOC Pediatria, Grande Ospedale Metropolitano Reggio Calabria Italy; ^11^ Dipartimento della Donna, del Bambino e di Chirurgia Generale e Specialistica, Università della Campania Luigi Vanvitelli Napoli Italy; ^12^ Dipartimento di Pediatria, Unità di Allergologia, Università di Messina Messina Italy; ^13^ Allergologia Pediatrica, Istituto Giannina Gaslini Genova Italy; ^14^ SODc Malattie Infettive AOU Meyer, Dipartimento di Scienze della Salute Università di Firenze Firenze Italy; ^15^ Dipartimento della Donna e del Bambino Università di Padova Padova Italy

**Keywords:** bronchiolitis, children, guidelines, survey, treatment

## Abstract

**Introduction:**

Significant variations in the management of bronchiolitis are often recorded, and, in parallel, to recommend a univocal clinical approach is challenging and still questioned. This study is aimed to evaluate the diagnostic and therapeutic management of bronchiolitis in children adopted by Italian pediatricians following the national guidelines.

**Material and Methods:**

A survey study was designed and carried out by sending an email an open‐ended questionnaire developed by an expert panel of the Scientific Board of the Italian Society of Pediatric Allergology and Immunology (SIAIP). Questions were designed according to the national intersociety consensus document on treatment and prevention of bronchiolitis in newborns and infants.

**Results:**

Overall, 234 pediatricians were taking part in the study. When diagnosing bronchiolitis, only 44.01% (103/234) of participants correctly followed the national guidelines. All participants (100%) would perform laboratory tests and/or radiological exams. 44.01% administered oxygen (O_2_) when O_2_ saturation was minor than 92%. About the therapeutic regimen, marked discrepancies between national guidelines and recorded answers were reported. Indications for hospital admission and discharge criteria were in line with the national guidelines.

**Conclusions:**

There is a significant practice variation in the management of acute bronchiolitis among Italian physicians. Some wrong attitudes need to be further discouraged, such use of diagnostic procedures and therapeutic approaches. Further research is urgently required to define the best management of patients with bronchiolitis and implement strategies to standardize care and improve the quality of care.

**Key messages statement**

*What does your study add?*

This study is aimed to evaluate the diagnostic and therapeutic management of bronchiolitis in children adopted by Italian pediatricians following the national guidelines.

*What are new insights?*

There is a significant practice variation in the management of acute bronchiolitis among Italian physicians. Some wrong attitudes need to be further discouraged, such use of diagnostic procedures and therapeutic approaches.

*What clinical implications does it bring?*

Further research is urgently required to define the best management of patients with bronchiolitis and implement strategies to standardize care and improve the quality of care.

## 1 INTRODUCTION

Bronchiolitis is the leading cause of hospital admission for respiratory disease among infants younger than 1‐year‐old. Clinically, children with bronchiolitis may present with a wide range of clinical symptoms, from mild to incipient respiratory failure.^1^ Diagnosis of bronchiolitis is based on the clinical history and a careful physical examination. However, clinical signs, such as rhinorrhea, cough, dyspnea, polypnea, apnea, crackles, wheezing, and feeding difficulties, are not specific, and they significantly overlap with symptoms and signs of other diseases.^2^ Bronchodilators, corticosteroids, antiviral therapy, and oxygen delivery methods have been suggested as pharmacological interventions in treating acute bronchiolitis; however, the evidence base for their benefit is poor.^3^, ^4^ Accordingly, a previous systematic review demonstrated heterogeneous behaviors regarding the adopted diagnostic and therapeutic approaches adopted among several European countries, based on the panel's expertise.^5^ Moreover, several aspects are still under debate, including the choice of the best diagnostic and therapeutic management as well as the caregivers' adherence to validated protocols. Therefore, significant variations in the therapeutic management of bronchiolitis between clinicians and hospitals are often recorded, and, in parallel, to recommend a univocal clinical approach, it is difficult and is still questioned.^3^, ^4^, ^5^

Considering the significant impact of these issues, the Italian Society of Paediatric Allergy and Immunology (SIAIP) conducted a national survey to evaluate the behaviors of Italian pediatricians in the diagnostic and therapeutic management of infants and children with acute bronchiolitis and their adherence to the current national recommendations^1^ (Table 1), also aiming to improve the management of bronchiolitis in children and standardize the behavior of physicians.

**Table 1:** The basic diagnostic and therapeutic management of bronchiolitis in accordance with the national recommendations

*Diagnostic criteria*
1. Onset with rhinorrhea and/or upper respiratory tract infections 2. First episode of respiratory distress associated with: crackles and/or wheezing, use of accessory muscles or lower chest wall retractions, low O_2_ saturation levels, high respiratory rate relative to age, skin color changes, nasal flaring, fever 3. Exposure to persons presenting with upper respiratory tract viral infections 4. Presentation during epidemic season
*Indications for hospital admission*
1. O_2_ saturation persistently lower than 90%–92%, entity of respiratory distress, presence of apnea 2. Dehydration 3. Moderate–severe bronchiolitis
Other important factors to take into consideration are:
1. Prematurity, gestational age <37 weeks, or birth age <6–12 weeks 2. Responsivity and alertness 3. Social and environmental factors 4. Presence of pre‐existing risk factors, comorbidities
*Diagnostic management*
1. Diagnosis of the disease is clinical 2. Neither laboratory radiological exams are usually indicated for the routine workup of infants suffering from bronchiolitis
*Treatment*
1. Oxygen therapy, if O_2_Sat < 90%–92% 2. Nebulized 3% hypertonic saline: safe and effective but further study is required 3. Inhaled Beta 2‐agonists: Not effective but the possibility of a therapeutic trial of salbutamol is suggested 4. Nebulized adrenaline: further studies are required 5. Systemic and nebulized steroids: not effective 6. Antibiotics: if bacterial coinfection 7. Respiratory physical therapy during acute phase of disease: not effective 8. Environment humidification: insufficient evidence
*Discharge criteria*
1. Patient does not need respiratory support 2. O_2_ saturation levels >92%–94% at ambient air 3. Stabilization of clinical presentation 4. Adequate oral intake of fluids and feeds (>75%) 5. Adequate social‐economic circumstances 6. If necessary, the possibility of obtaining pediatric health care assistance locally

## 2 MATERIALS AND METHODS

This study is aimed to evaluate the diagnostic and therapeutic management of bronchiolitis in children adopted by Italian pediatricians according to the national guidelines.^1^

The study was proposed in September 2019 by the Scientific Board of the SIAIP. A specific website was prepared for the study.

The survey was announced and firstly administered to all the pediatricians attending the “Opinoni a Confronto” Annual Meeting, held in Pavia, Lombardia (Italy) in October 2019. Overall, 100 questionnaires were distributed and full‐filled. After that, the study was carried out by sending by email an open‐ended questionnaire. Participation in the study was voluntary.

### 2.1 Structured questionnaire

The questionnaire included an explanatory cover letter reporting the aim of the survey. Experts designed the questions in the field (S. M., A. L., G. L. M., and E. C.).

The structured questionnaire was prepared as a specific form to be fulfilled online (closed‐ and open‐ended format questions) on the website. Responses were anonymous, but professional information (university pediatrician, hospital pediatrician, primary care pediatrician, or pediatric resident) was requested. The questionnaire included 10 questions in a multiple‐choice format. Specifically, the questionnaire contained questions on diagnostic criteria, diagnostic and therapeutic management of bronchiolitis in children aged <24 months. One or more answers for each question were provided.

The survey was administered in Italian and translated into English for publication. The English version of the questionnaire is enclosed in Supporting Information Appendix A.

### 2.2 Statistical analysis

The results were given as absolute numbers and percentages. The percentages of responses to the questions have been calculated on the total of the participants. SPSS software package (SPSS 11.5) was used, and *p* < .05 was considered as statistically significant.

## 3 RESULTS

The answers to the survey by the participating Italian pediatricians are summarized in Supporting Information Appendix B (a–i and l).

### 3.1 Participants' characteristics

Overall, 234 pediatricians (27.7% of physicians registered to the Congress) returned the questionnaire. The completion rate was 100%.

The average time taken to answer a question was 5 min.

The three Italian macro‐regions were represented as follows: North 136/234, Centre 55/234, and South 43/234.

Most of the participants in the survey were residents in pediatrics (39.74%, 93/234) followed by hospital pediatricians (30.34%, 71/234), family pediatricians (18.8%, 44/234), and university pediatricians (11.11%, 26/234).

Baseline characteristics of the cohort examined are summarized in Table 2.

**Table 2:** Baseline characteristics of the examined cohort

Macro‐regions	North: Aosta Valley, Piedmont, Liguria, Lombardy, Emilia‐Romagna, Veneto, Friuli‐Venezia Giulia and Trentino‐Alto Adige/Südtirol
	Centre: Lazio, Marche, Tuscany, and Umbria
	South: Molise, Abruzzo, Campania, Basilicata, Calabria, Puglia, Sicily, and Sardinia.
Participants	Residents in pediatrics
	Hospital pediatricians
	Family pediatricians
	University pediatricians
Years of practice	<5 years for:
	Residents in pediatrics
	More than 5 years for:
	Hospital pediatricians
	Family pediatricians
	University pediatricians
Number of inpatients versus outpatients	237 versus 94

### 3.2 Epidemiological findings

The mean number of patients reporting physician‐diagnosed bronchiolitis was <25 (34.18%, 80/234), 25–50 (32.47%, 76/234), >50 (33.33%, 78/234).

### 3.3 Diagnostic criteria

When diagnosing bronchiolitis, 44.01% (103/234) of participants correctly included the presence of all the following criteria: rhinorrhea and/or upper respiratory tract infections; first episode of respiratory distress featured by crackles and/or wheezing, nasal flaring, tachypnea, accessory muscles use or chest retractions, decrease in O_2_ saturation, skin color changes, fever; contact with individuals presenting with upper respiratory tract viral infections; and the onset of symptoms during the epidemic season. For 5.5% (13/234) of participants, diagnostic criteria included rhinorrhea and/or upper respiratory tract infections. For 33.76% (79/234) of participants, diagnostic criteria included the first episode of respiratory distress featured by crackles and/or wheezing, accessory muscles use or chest retractions, decrease in O_2_ saturation, tachypnea, skin color changes, nasal flaring, fever. For 6% (14/234) of participants, diagnostic criteria included contact with individuals presenting with upper respiratory tract viral infections. For 10.68% (25/234) of participants, diagnostic criteria included clinical presentation during the epidemic season.

### 3.4 Approach to the no well‐appearing child

Although neither laboratory nor radiological exams are supported for the routine workup of children affected by bronchiolitis, the proportion of participants who would obtain complete blood count was 43.8%. 2.37% would perform blood culture; 85.21% blood gas analysis, 26.63% serum electrolytes, 7.69% glycemia, 46.75% C‐reactive protein (C‐RP), 70.41% polymerase chain reaction on the nasal swab, and 13.61% chest X‐ray.

### 3.5 Indications for oxygen administration

A total of 20.08% (47/234) of participants would administer oxygen (O_2_), whether O_2_ saturation is persistently lower than 90% and 35.9% (84/234) when O_2_ saturation ranges between 90% and 92%. Conversely, not following the guideline recommendations, 15.81% (37/234) when O_2_ saturation ranges between 92% and 94%; and 28.2% (66/234) when O_2_ saturation is persistently lower than 94%.

### 3.6 Administration of medications

Marked discrepancies between national guidelines and recorded answers were reported. The most common treatment administered for bronchiolitis was high flow oxygen therapy (81.66%) followed by oxygen therapy with nasal cannula or face mask (68.05%), hypertonic solution (49.70%), inhaled short‐acting β‐agonists (SABA) (39.64%), inhaled epinephrine (21.30%), an inhaled corticosteroid (ICS) (17.16%), systemic CS (64.52%), respiratory physiotherapy (8.88%), antibiotics (4.73%), intravenous SABA (0.59%). Among pediatricians using systemic CS, betamethasone was the most common CS administered (64.52%), followed by methylprednisone (16.66%), prednisolone (7.26%), beclometasone dipropionate (4.7%), and dexamethasone (4.7%). In line with national guidelines, only 16.66% of the participants did not administer systemic CS.

### 3.7 Indications for hospital admission

The indications for hospital admission included poor feeding or dehydration (98.82%), comorbidities (98.22%), need for supplemental oxygen therapy (96.45%), poor social circumstances (82.25%), prematurity (82.25%), presence of apnea (81.66%), infants <3 months of age (79.29%), cyanosis (78.11%), tachypnea (96.44%), fever (>38°C) (44.97%), uncertain of diagnosis (29.59%), and unreliable parents (81.66%).

### 3.8 Discharge criteria

In line with national guidelines, the discharge criteria adopted by interviewed physicians were: improvement in clinical conditions (47.93%), adequate oral feeding (92.31%), improved respiratory effort (92.90%), improved O_2_ saturations (SaO_2_ > 97%) (52.07%), improved O_2_ saturations (SaO_2_ > 94%) (37.87%), improved O_2_ saturations (SaO_2_ > 92%) (5.33%), carer ability (78.70%), adequate social circumstances (18.34%), possibility to arrange follow‐up (67.46%).

## 4 DISCUSSION

Significant variations in the management of bronchiolitis are often recorded, and, in parallel, to recommend a univocal clinical approach is challenging and still questioned. This study aimed to evaluate the diagnostic and therapeutic management of bronchiolitis in children adopted by Italian pediatricians according to the national guidelines. Our survey showed a significant practice variation in the management of acute bronchiolitis among Italian physicians.

Nationally developed evidence‐based guidelines are required to reduce practice variation, minimize nonevidence‐based practices, and promote cost‐effective standardization of care.^6^ Nevertheless, a significant practice variation persists, and it has been previously reported that several guideline recommendations have been poorly incorporated into the real clinical settings after their development and dissemination.^7^, ^8^ Accordingly, several international studies have detected substantial variations in the use of medication for infant bronchiolitis between and within countries^9^, ^10^ A significant practice variation in the monitoring, treatment, and discharge of the children hospitalized with bronchiolitis among physicians across Italian pediatric hospitals has also been founding.^5^ Therefore, given the high risk of inappropriate and unnecessary medicalization and hospitalizations of patients with bronchiolitis and, consequently, the bronchiolitis‐related high‐financial burden, it is important to examine the diagnostic and therapeutic interventions administered in infants presenting with bronchiolitis. In this regard, the SIAIP conducted a national survey to evaluate the behaviors of Italian pediatricians in the diagnostic and therapeutic management of infants and children with acute bronchiolitis and their adherence to the current national recommendations. To the best of our knowledge, a similar investigation had not yet been conducted in Italy, making this study essential for understanding the Italian management of patients suffering from bronchiolitis. This information would also clarify the use of evidence‐based supportive therapies compared with those that are not evidence‐based, aiming to improve the management of bronchiolitis in children and standardize physicians' behavior.

The diagnosis of bronchiolitis is based on clinical history and physical examination.^1^ In accordance with the national guidelines,^1^ <50% of participants agreed on the diagnostic criteria, and the most common criteria for bronchiolitis diagnosis were rhinorrhea and/or upper respiratory tract infections; the first episode of respiratory distress featured by crackles and/or wheezing, nasal flaring, tachypnea, accessory muscles use or chest retractions, decrease in O_2_ saturation, skin color changes, fever; contact with individuals presenting with upper respiratory tract viral infections; and the onset of symptoms during the epidemic season. Marked discrepancies could be observed between the remaining respondents as well as inappropriateness in the answer.

National guidelines include as criteria for hospital admission the following: O_2_ saturation persistently lower than 90%–92%, the entity of respiratory distress, presence of apnea, dehydration, and moderate to severe bronchiolitis.^1^ Other important factors that should be taken into consideration are prematurity (defined as gestational age <37 weeks or birth age <6–12 weeks), consciousness and alertness, poor hydration and feeding (defined as <50% of usual fluid intake in preceding 24 h), social and environmental determinants, and presence of pre‐existing risk factors.^1^

The decision to hospitalize a child affected by bronchiolitis is a complex process impacted by the course of the illness and other clinical, sociocultural and geographic factors as well as by the possibility to access to the follow‐up.^1^, ^11^ Moreover, the local culture of care can influence the decision‐making process, irrespective of disease severity. Thus, although inpatient observation is recommended for selected infants affected by bronchiolitis, physicians are more likely to hospitalize infants with milder bronchiolitis, taking improper advantage of the use of inpatient resources. On the other hand, the variability in admission criteria among guidelines may alter unavoidably the hospitalization criterion. In this regard, it must be considered that some institutions have protocols that require the ordering of diagnostic tests (e.g., complete blood count, chest X‐ray) on admission. However, a priori reasons to support this clinical practice are not currently supported by evidence‐based medicine.^12^ Moreover, this practice could lead both to perform costly and unnecessary testing/procedure cascade, and inappropriate outpatient/inpatient antibiotic prescriptions.

The administration of nonrecommended interventions in our study occurred at a moderate‐to‐high rate, as follows: inhaled epinephrine (21.30%), ICS (17.16%), systemic CS (64.52%), respiratory physiotherapy (8.88%), antibiotics (4.73%), and intravenous SABA (0.59%). The current literature does not recommend the diagnostic testing for children with bronchiolitis, except for conducting epidemiological studies. Moreover, very few data support the impact of testing on patient outcomes and quality of care, and they do not provide clear indications for such testing or the impact of testing on relevant patient outcomes.^1^, ^5^, ^13^ Moreover, the overuse of instrumental diagnostic tests does not comply with patient safety and weighs on the healthcare economy.

Elsewhere, the guidelines developed by a panel of experts aim to minimize overtreatment by recommending against the use of noneffective interventions.^14^, ^15^, ^16^, ^17^, ^18^, ^19^, ^20^ Nevertheless, authors of inpatient bronchiolitis studies published after the 2006 American Academy Pediatrics bronchiolitis guideline have found disappointingly high use of the non recommended resources.^21^, ^22^ We found a surprising number of children were treated with medications. Following the Italian guidelines,^1^ supplemental oxygen (O_2_) should be administered if O_2_ saturation levels are persistently below 90%–92% at ambient air.^1^, ^23^

A recent trial has suggested that outcomes may not differ significantly when an O_2_ saturation target of ≥90% is used.^24^ High‐flow oxygen therapy with humidified and heated oxygen (high‐flow nasal cannula) should be considered whether an increased respiratory effort is occurring. However, different O_2_ saturation thresholds are recommended for hospital management and for starting O_2_ therapy. Thus, it is reasonable to hypothesize that both the difference in the evidence provided by the literature as well as context‐specific factors may be key reasons for the differences in clinical practice. In children with bronchiolitis who cannot maintain oral hydration intravenous or nasogastric fluids may be considered.^1^, ^23^ There is sparse evidence supporting the routine use of hypertonic solution, nebulized adrenaline, salbutamol, respiratory physiotherapy, systemic or inhaled or systemic CS, and antibiotics.^1^, ^23^ However, authors support the use of hypertonic saline to decrease airway edema and improve mucociliary clearance in infants.^1^, ^23^ Unlike other respiratory diseases in which CS show a beneficial effect, their use does not improve the short‐ and long‐term prognosis in infants with bronchiolitis.^1^, ^23^

Similarly, the association of systemic CS with epinephrine or SABA does not produce a significant benefit.^1^, ^23^ The administration of bronchodilators in managing bronchiolitis is still contentious and, although evidence is against their routine use, several guidelines still recommended a trial of bronchodilators in treating infants with acute bronchiolitis.^5^ Despite the absence of evidence suggesting a benefit of antibiotic use in acute bronchiolitis, their prescription in primary care settings is commonly reported.^1^, ^23^ Evidence supporting the use of nebulized adrenaline, antivirals, antileukotrienes, and manual chest physiotherapy (such as vibration and percussion) during the early stages of bronchiolitis is insufficient.^1^

Surprisingly, regarding the discharge criteria, the healthcare professional behavior was in line with the national guidelines.^1^ An appropriate discharge requires evaluation of multiple factors including medical (clinical conditions, adequate feeding, improved respiratory effort), psychosocial (carer ability), logistic (possibility to arrange follow‐up), and economic (adequate social circumstances) considerations. Discharge planning must involve both the health care team and patient/family caregivers to develop a patient‐centered plan ensuring that the patient is safely discharged home and minimizing the risk of adverse events and/or unplanned readmissions.^23^, ^25^

Except for the data on the discharge criteria, we formulated different hypotheses to explain the striking differences between our study's findings and the guideline‐related recommendations of nonintervention. Acute bronchiolitis significantly causes pressures in health service and throughout the region. Probably, the parental expectation “to do something” might be in part responsible for these results, also increasing the local practice differences.‍ In a previous study, authors reported that bronchiolitis hospitalization caused significant emotional, physical, and organizational consequences for parents and siblings persisting up to 3 months after hospital discharge.^26^ Moreover, it is possible that primary care pediatricians, who may have limited experience with managing acute bronchiolitis in advanced stage of disease, are influenced wrongly by the medical literature showing benefit of SABA and/or ICS in other respiratory diseases.^23^, ^25^ Furthermore, there is evidence that exposure to a few cases of patients with bronchiolitis is associated with the increased use of investigations and medications.^27^, ^28^ Lastly, the difficulty and/or the impossibility to arrange follow‐up care can affect disease management.

### 4.1 Limits of the study

The survey design generates findings entirely based on recall over a previous calendar year. However, given the fact there has been no national or local implementation of a standardized bronchiolitis guideline, we can exclude with reasonable certainty that a significant change in clinical practice has occurred since the time our data were recorded. Furthermore, what physicians report and what they do in real clinical practice may often vary.

Data were only collected from 234 pediatricians, but this denominator can not be definitively representative of all nations. Moreover, small sample sizes in some sites have limited our ability to investigate site‐to‐site variation. On the other hand, the solid response rate and the broad representation of sites across the whole country lend weight and credibility to our results.

Finally, relevant conclusions for other countries cannot be drawn from our results; however, substantial variability in medicines prescribed for acute bronchiolitis has been similarly reported in different countries.^10^

## 5 CONCLUSIONS

There is a significant practice variation in the monitoring, treatment, and discharge of children suffering from acute bronchiolitis among Italian physicians. Future research is urgently required to define the best management of patients with bronchiolitis, optimize the use of therapeutic resources, and implement strategies to standardize care and improve the quality of care.

## CONFLICT OF INTERESTS

The authors declare that there are no conflict of interests.

## AUTHOR CONTRIBUTIONS

Sara Manti, Amelia Licari, Elena Chiappini, and Gianluigi Marseglia designed the study. Ilaria Brambilla, Carlo Caffarelli, Mauro Calvani, Fabio Cardinale, Giorgio Ciprandi, Giovan B. Pajno, and Claudio Cravidi contributed to the data collection. Sara Manti, Amelia Licari, and Elena Chiappini wrote the initial draft of the manuscript. Marzia Duse, Alberto Martelli, and Domenico Minasi performed a critical revision of the manuscript. Amelia Licari, Fabio Cardinale, and Giorgio Ciprandi analysed the data. Michele Miraglia Del Giudice, Maria A. Tosca, and Eugenio Baraldi offered precious technical advice on how the study might be improved. Gianluigi Marseglia supervised the study. All authors provided substantial contributions to the conception or design of the work, or the acquisition, analysis, or interpretation of data for the paper, revised the manuscript for important intellectual content, approved the final version, and agreed to be accountable for all aspects of the work.

## Supporting information

Supporting information.

Supporting information.

## Data Availability

Research data are not shared.
