# Supplementary material for: Agreements and controversies of national guidelines for bronchiolitis: Results from an Italian survey
Source: Immun Inflamm Dis. 2021 Oct 22;9(4):1229–36. doi: 10.1002/iid3.451 (PMC8589388; doi:10.1002/iid3.451)
Supplement: Supplementary file 1 — Supporting information. [file IID3-9-1229-s001.docx]

**Appendix**

Appendix A**.** English version of the questionnaire administered.

| 1. Where do you work? 2. Northen Italy 3. Central Italy 4. Southern Italy |
| --- |
| 1. Describe your current position 2. University paediatricians 3. Hospital paediatricians 4. Family paediatricians 5. Residents in paediatrics |
| 1. How many cases of bronchiolitis did you diagnose last year? 2. <25 3. 25-50 4. >50 |
| 1. Which of the following diagnostic criteria do you take into account to diagnose bronchiolitis? 2. Rhinorrhea and/or upper respiratory tract infections 3. First episode of respiratory distress associated with crackles and/or wheezing, use of accessory muscles or lower chest wall retractions, low O2 saturation levels, high respiratory rate relative to age, skin color changes, nasal flaring, fever 4. Exposure to persons presenting with upper respiratory tract viral infections 5. Clinical presentation during epidemic season 6. All the above mentioned criteria |
| 1. How is bronchiolitis diagnosed?   (more than one answer possible)   1. Complete blood count 2. Blood culture 3. Blood gas analysis 4. Serum electrolytes 5. Glycemia 6. C-reactive protein 7. Polymerase chain reaction on nasal swab 8. Chest X-ray |
| 1. At what oxygen saturation is oxygen needed? 2. SaO2<90% 3. SaO2 90-92% 4. SaO2 92-94% 5. SaO2 >94% |
| 1. How do you manage bronchiolitis?   (more than one answer possible)   1. High flow oxygen therapy 2. Oxygen therapy with nasal cannula or face mask 3. Hypertonic solution 4. Inhaled short-acting beta-agonists 5. Inhaled epinephrine 6. Inhaled corticosteroid 7. Systemic corticosteroid 8. Respiratory physiotherapy 9. Antibiotics 10. Intravenous short-acting beta-agonists |
| 1. If you are using systemic corticosteroid, which type do you administer? 2. betamethasone 3. metylprednisone 4. prednisolone 5. beclometasone dipropionate 6. dexamethasone |
| 1. Which of the following criteria for hospital admission do you adopt?   (more than one answer possible)   1. Poor feeding or dehydration 2. Comorbidities 3. Prematurity 4. Presence of apnea 5. Infants less than 3 months of age 6. Cyanosis 7. Tachypnea 8. Fever (>38°C) 9. Uncertain of diagnosis 10. Unreliable parents |
| 1. Which of the following criteria for hospital discharge do you adopt? 2. Improvement in clinical conditions 3. Adequate oral feeding 4. Improved respiratory effort 5. Improved O2 saturations (SaO2 >97%) 6. Improved O2 saturations (SaO2 >94%) 7. Improved O2 saturations (SaO2 >92%) 8. Carer ability 9. Adequate social circumstances 10. Possibility to arrange follow-up |
