# Supplementary material for: Agreements and controversies of national guidelines for bronchiolitis: Results from an Italian survey
Source: Immun Inflamm Dis. 2021 Oct 22;9(4):1229–36. doi: 10.1002/iid3.451 (PMC8589388; doi:10.1002/iid3.451)

**Appendix B (a, b, c, d, e, f, g, h, i, l).** Answers provided by the participating Italian pediatricians to the survey. (a, b) Participants' characteristic (data are expressed in absolute value). (c) Mean number of patients reporting physician-diagnosed bronchiolitis (data are expressed in absolute value). d) Diagnostic criteria: A: rhinorrhea and/or upper respiratory tract infections; B: first episode of respiratory distress featured by crackles and/or wheezing, accessory muscles use or chest retractions, decrease in O2 saturation, tachypnea, skin colour changes, nasal flaring, fever; contact with individuals presenting with upper respiratory tract viral infections; and onset of symptoms during the epidemic season; C: persons presenting with upper respiratory tract viral infections; D: clinical presentation during the epidemic season; E: all the previous criteria (data are expressed in percentage). e) Approach to the no well-appearing child: A: complete blood count, B: blood culture; C: blood gas analysis; D: serum electrolytes; E: glycemia; F: C-reactive protein (C-RP); G: polymerase chain reaction (PCR) on the nasal swab; H: chest X-ray (data are expressed in percentage). f) Indications for oxygen administration (data are expressed in percentage). g) Administration of medications: A: high flow oxygen therapy; B: oxygen therapy with nasal cannula or face mask; C: hypertonic solution; D: inhaled short-acting beta-agonists; E: inhaled epinephrine; F: inhaled corticosteroid; G: systemic corticosteroid; H: respiratory physiotherapy; I: antibiotics; L: intravenous short-acting beta-agonists; M: antivirals; N: antileukotrienes; O: systemic epinephrine (data are expressed in percentage). h) The most common corticosteroids administered: A: betamethasone, B: methylprednisone, C: prednisolone, D: beclometasone dipropionate, E: dexamethasone; F: none (data are expressed in percentage). i) Indications for hospital admission: A: infants less than 3 months of age; B: prematurity; C: respiratory rate >30 breaths/minute; D: respiratory rate >50 breaths/minute; E: respiratory rate >60 breaths/minute; F: poor feeding or dehydration; G: cyanosis; H: apnea; I: comorbidities; L: poor social circumstances; M: uncertain of diagnosis; N: need for supplemental oxygen therapy; O: fever; P: unreliable parents (data are expressed in percentage). l) Discharge criteria: A: SaO2 >97%, B: SaO2 >94%; C: SaO2 >92%; D: improved respiratory effort; E: adequate oral feeding; F: adequate social circumstances; G: carer ability; H: possibility to arrange follow-up; I: improvement in clinical conditions (data are expressed in percentage).


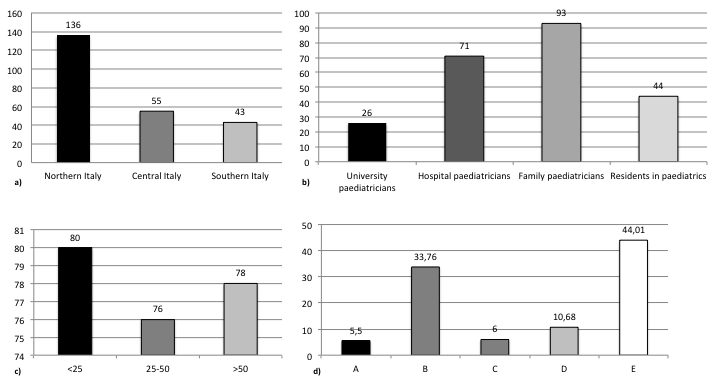


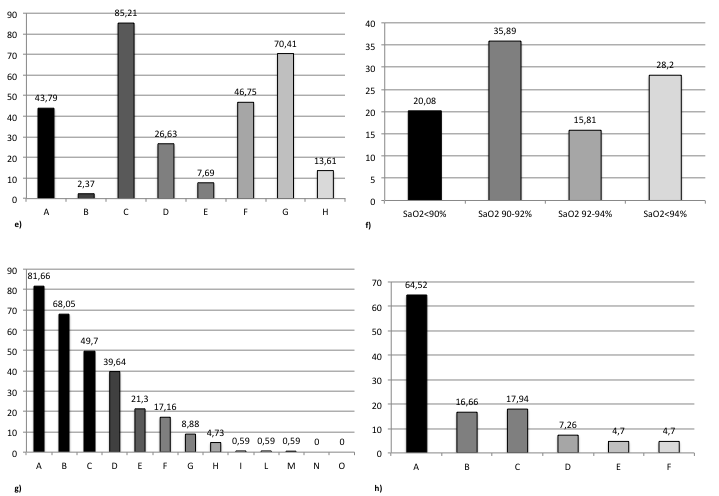


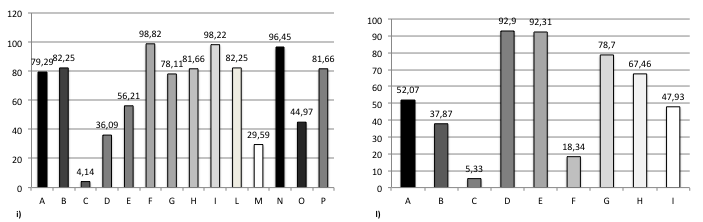

Supplement: Supplementary file 2 — Supporting information. [file IID3-9-1229-s002.docx]
